# Anomalous origin of the left circumflex artery from the right coronary artery: a case report

**DOI:** 10.1186/1757-1626-1-336

**Published:** 2008-11-19

**Authors:** Sotiris C Plastiras, Ourania S Kampessi, Maria Gotzamanidou, Panayiotis Kastanis

**Affiliations:** 1 Department of Pathophysiology, University of Athens Medical School, Laiko Hospital, Greece; 2 Department of Clinical Therapeutics, Invasive Cardiology Unit, University of Athens Medical School, Alexandra Hospital, Greece

## Abstract

**Introduction:**

Coronary artery anomalies are found in 0.6% to 1.55% of patients who undergo coronary angiography, and the increasing use of diagnostic coronary angiography is uncovering even more such abnormalities. We present a very unusual case of an anomalous origin of the left circumflex coronary artery (LCx) from the proximal right coronary artery (RCA).

**Case presentation:**

We present a case of a 45-year-old-man with a recent history of a non ST elevation myocardial infarction. The coronary angiography reveals an ectopic left circumflex coronary artery from the right coronary artery. In this report we attempted to highlight the rarity of this coronary anatomy.

**Conclusion:**

Anomalous origins of the coronary artery are rare, but may cause myocardial ischemia and sudden death. Thus, their reliable identification is a matter of paramount importance possibly evaluating the effects of therapeutic intervention.

## Introduction

The presence of anomalous coronary arteries is observed about 1% of patients undergoing cardiac catheterization. However, their identification is crucial to the management of the patient with associated coronary artery disease. This report presents a very unusual case of an anomalous origin of the left circumflex coronary artery (LCx) from the proximal right coronary artery (RCA) in a patient presented with a non ST elevation myocardial infarction of the inferior wall.

## Case presentation

**Figure 1:**
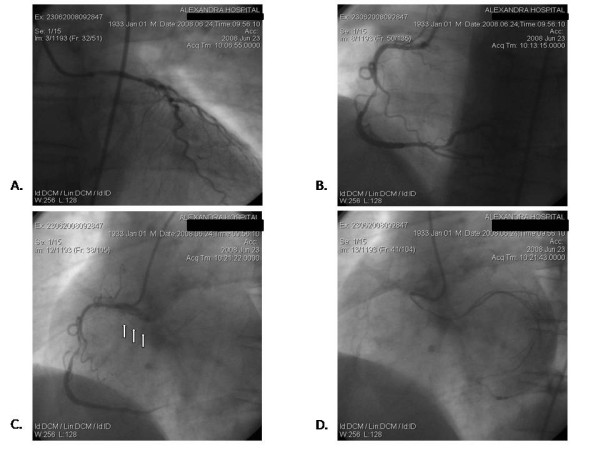
**(A) The left anterior descending artery (LAD).** (B, C) The right coronary artery (RCA); note the stenotic lesion in the middle of RCA and the anomalous origin of the left circumflex artery (LCx) from the proximal RCA. (D) The ectopic LCx.

A 45-year-old man was admitted to our hospital with a recent history of chest pain. He had no specific past medical history, but he was a smoker. The electrocardiogram reveals mildly depression of ST in the leads of the inferior wall. The troponin and the remaining cardiac enzymes were elevated. He was hemodynamically stable with a blood pressure approximately 120/80 mmHg. The patient received dual antiplatelet therapy (aspirin 100 mg/day and clopidogrel 300 mg once and after at a dose of 75 mg/day), intravenously glycoprotein IIbIIIa inhibitors and heparine for 48 hours; the remaining therapy was a statin, an angiotensin converting enzyme inhibitor (ramipril) and a beta blocker (metoprolol). Afterwards, he underwent coronary angiography to evaluate coronary artery disease because of his recent history of non ST elevation myocardial infarction of inferior wall. In this patient, the mildly stenosed LCx coexists with a stenosed RCA, causing a non-ST elevation coronary syndrome (Figure 1). He underwent successfully coronary angioplasty of the RCA, which was the responsible artery for the non ST elevation myocardial infarction.

## Conclusion

The ectopic origin of the LCx is a well-recognized variant, which is considered the most common coronary anomaly and can be found in approximately 0.37% to 0.7% of all patients. The anomalous LCx most commonly arises from a separate ostium within the right sinus, or as a proximal branch of the RCA [1]. Although this anomaly is classified as benign and asymptomatic, and a few cases of sudden death, myocardial infarction, and angina pectoris in the absence of atherosclerotic lesions have been reported [2]. The technical experience reported in the literature concerning angioplasty, if needed, in patients with anomalous origin of the left circumflex artery is limited. Balloon angioplasty seems to be a favorable approach for revascularization in these vessels, and major determinants of successful angioplasty are angiographic knowledge of their course and structure, appropriate selection of guiding catheter, and the possibility of advancing the balloon into the anomalous vessel [3,4].

## Consent

Written informed consent was obtained from the patient's next-of-kin for publication of this case report and accompanying images. A copy of the written consent is available for review by the Editor-in-Chief of this journal.

## Competing interests

The authors declare that they have no competing interests.

## Authors' contributions

OK and MG analyzed and interpreted the patient data regarding his hospitalization. SP and PK performed the coronary angiography, and were the major contributors in writing the manuscript. All authors read and approved the final manuscript.
